# Research progress on multidimensional intervention strategies for hyperuricemia: Western medicine, Traditional Chinese Medicine, and emerging therapies

**DOI:** 10.3389/fendo.2025.1722245

**Published:** 2025-12-19

**Authors:** Xinze Li, Zilong Chen, Yanzhao Zhang, Chunyang Fan, Lulu Chen, Xiangman Xu, Jianying Chang, Wei Qiang, Hongwei Jiang, Chuanxin Liu

**Affiliations:** Endocrinology and Metabolism Center, The First Affiliated Hospital, and College of Clinical Medicine of Henan University of Science and Technology, Luoyang, China

**Keywords:** hyperuricemia, uric acid-lowering drugs, URAT1 inhibitors, xanthine oxidoreductase inhibitors, uric acid transporter modulators, Traditional Chinese Medicine, mazdutide, washed microbiota transplantation

## Abstract

Hyperuricemia is a metabolic disease caused by purine metabolism disorders. In recent years, its incidence has been increasing year by year and showing a trend of rejuvenation. It is closely associated with various health issues such as gout, kidney damage, and cardiovascular diseases. Therefore, standardizing and updating its treatment strategies holds significant clinical importance. This article systematically reviews the current various intervention methods and research status for the treatment of hyperuricemia: In the field of Western medicine, it deeply analyzes the efficacy, mechanism of action, and clinical limitations of drugs that promote uric acid excretion (such as benzbromarone and dotinurad), drugs that inhibit uric acid synthesis (such as allopurinol, febuxostat, and topiroxostat), and drugs that promote uric acid hydrolysis (such as pegloticase and rasburicase). It focuses on elaborating the research breakthroughs of URAT1 inhibitor derivatives and the new drug SHR4640. In the field of Traditional Chinese Medicine (TCM), from three aspects of single-herb monomers, compound prescriptions, and external treatment methods, it reveals their advantages in reducing uric acid through multiple mechanisms, including inhibiting xanthine oxidase (XOD), regulating uric acid transporters such as URAT1, GLUT9, and OATs, and improving intestinal homeostasis, with particular emphasis on the structure-activity relationship of flavonoids. At the same time, it details the action pathways and clinical evidence of emerging therapies such as SGLT2 inhibitors, the GLP-1/GCG dual-receptor agonist Mazdutide, probiotics, and washed microbiota transplantation (WMT). By summarizing mechanistic insights, clinical progress, and translational prospects, this review aims to inform the development of individualized and integrative therapeutic strategies for hyperuricemia.

## 1 Overview of hyperuricemia

**Figure 1:**
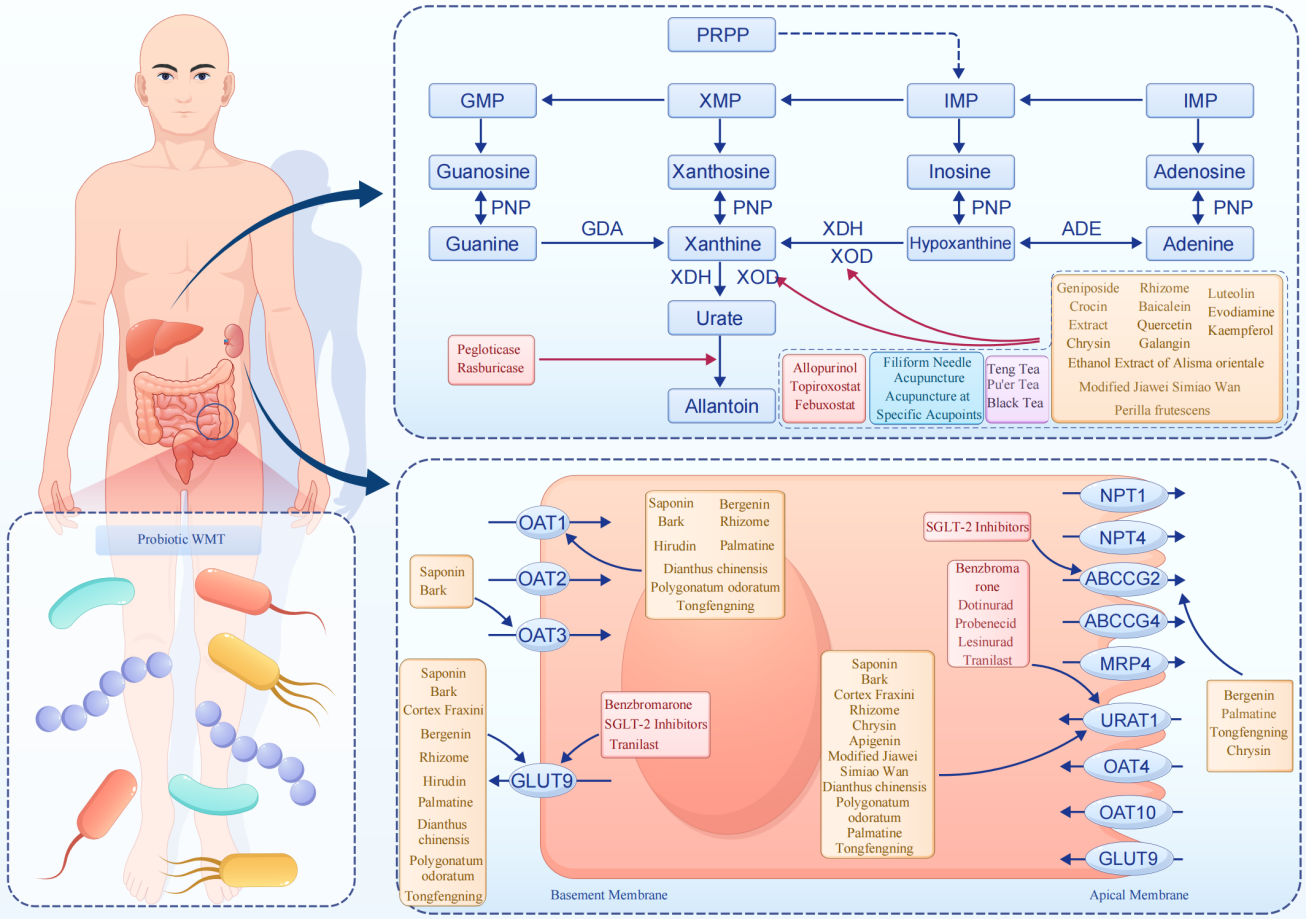
The purine metabolism process of hyperuricemia and the mechanism of action of various intervention methods.

The concentration of uric acid in the blood mainly depends on the balance between the production and excretion of the end-products of purine metabolism. The causes of hyperuricemia mainly include genetic factors, dietary factors, drug effects, and lifestyle (1, 2). The pathogenic factors of hyperuricemia include genetics, diet, drugs, and lifestyle: Primary hyperuricemia caused by genetic factors is related to abnormal activity of purine metabolism enzymes; Secondary hyperuricemia is mostly caused by systemic diseases or drugs that inhibit uric acid excretion; Although high-purine foods in the diet (such as meat, seafood, alcohol, etc.) only account for 20% of the source of uric acid, excessive intake can aggravate the increase of uric acid; In addition, obesity and metabolic syndrome are also associated with an increased risk of hyperuricemia. With the economic development and changes in lifestyle, the incidence of hyperuricemia has been continuously increasing and showing a trend of rejuvenation, and it has become the second most common metabolic disease after diabetes. Abnormal elevation of serum uric acid is not only directly related to gout and kidney diseases, but also increases the risk of cardiovascular diseases, diabetes, hypertension, and dyslipidemia (3). Therefore, effective management of serum uric acid levels is of great clinical significance for preventing related complications and improving the prognosis of patients. The overall metabolic process and corresponding intervention mechanisms are illustrated in **Figure 1**.

## 2 Research progress of uric acid-lowering drugs promoting uric acid excretion

The kidney is the main organ for uric acid excretion. The reabsorption of uric acid in the renal tubule depends on the synergistic effect of a variety of transporters, including urate transporter 1 (URAT1), glucose transporter 9 (GLUT9), organic anion transporter 4 (OAT4), and organic anion transporter 10 (OAT10), among which URAT1 plays a major role (4). Regulating the activity of these transporters, especially URAT1, can affect the excretion of uric acid and is crucial for maintaining the balance of serum uric acid. The inhibition of URAT1 is an effective strategy to promote uric acid excretion, and the inhibition of other transporters may have a synergistic effect with URAT1 inhibition in reducing uric acid levels.

### 2.1 Benzbromarone

As a benzofuran derivative, benzbromarone mainly reduces serum uric acid levels and promotes the dissolution of monosodium urate (MSU) crystals by targeting URAT1 and GLUT9 to inhibit the reabsorption of uric acid in the renal tubule. Although benzbromarone is still a common choice for patients with insufficient uric acid excretion in China, clinical attention should be paid to the individual differences in its hepatotoxicity. Studies have pointed out that cases of fulminant hepatic necrosis caused by this drug in Caucasians have led European and American guidelines to classify it as a second-line drug. However, a domestic retrospective study showed that when 50 mg/d benzbromarone was combined with 20 mg febuxostat to treat gout with insufficient renal excretion, although the uric acid-lowering compliance rate (89.2%) was significantly higher than that of the single-drug group, liver function indicators (ALT, AST) should be monitored every 4 weeks, especially for patients with non-alcoholic fatty liver (5). In recent years, studies have found that the selectivity of benzbromarone for URAT1 is relatively poor. Researchers have improved the selectivity of benzbromarone derivatives for URAT1 through structural modification and reduced their hepatotoxicity. Compound 51a, as the most promising URAT1 inhibitor, not only maintains the inhibitory activity similar to that of benzbromarone, but also reduces the inhibitory effect on other transporters, showing better safety (6). In addition, the combination of benzbromarone and allopurinol can significantly improve the therapeutic effect and reduce the frequency of gout attacks, and this concept has been applied to the combined treatment of other xanthine oxidase inhibitors and uric acid excretion-promoting drugs (7). Involving 150 patients with hyperuricemia complicated with gout showed that after 8 weeks of treatment with 50 mg/d benzbromarone combined with 0.3 g tid sodium bicarbonate, the total effective rate reached 92% (significantly higher than 74.67% in the group using sodium bicarbonate alone), and the decreases in serum uric acid (UA), serum creatinine (Scr), and urea levels were more significant, and the number of tophi and VAS pain scores decreased more obviously (8).

### 2.2 Dotinurad

Dotinurad is the most successful benzbromarone derivative, which increases uric acid excretion by highly selectively inhibiting URAT1. Compared with other similar inhibitors, dotinurad shows higher selectivity and more significant inhibitory effect (9). The results of a phase III clinical study previously conducted in Japan showed that in patients with hyperuricemia with or without gout treated with 4 mg dotinurad, the proportion of patients with serum uric acid level ≤ 6 mg/dL at 58 weeks reached 100%, and long-term use had no significant impact on renal function and did not cause clinically relevant liver function abnormalities (10). In addition, dotinurad has also shown a role in improving renal function in patients with chronic kidney disease, suggesting its potential application value in patients with hyperuricemia complicated with renal insufficiency (11). It is worth noting that dotinurad not only shows excellent effects in regulating uric acid levels, but also its cardioprotective effect provides a new perspective for its application in the treatment of metabolic heart disease. In a study, the use of dotinurad significantly reduced cardiac fibrosis and inflammatory response in obese mice fed a high-fat diet. By reducing the expression of URAT1 and inhibiting the activation of the MAPK pathway, dotinurad reduced myocardial cell apoptosis, oxidative stress, and inflammatory response induced by saturated fatty acid palmitic acid, thereby alleviating cardiac fibrosis. This indicates that dotinurad has a direct protective effect on the heart and provides a new direction for the treatment of metabolic heart disease (12).

### 2.3 Probenecid

Probenecid is a classic non-selective URAT1 inhibitor that has been used clinically for decades. Probenecid inhibits the renal secretion of organic acids such as penicillins and cephalosporins, an effect demonstrated *in vitro* using human embryonic kidney 293 cells stably expressing organic anion transporters OAT1 and OAT3 that mediate tubular organic acid excretion (13). Studies have shown that in addition to reducing serum uric acid levels by inhibiting the uric acid transporter URAT1, probenecid may also enhance its uric acid-lowering effect by affecting other transporters such as OATs (organic anion transporters). However, due to extensive drug-transporter interactions characterized in cellular models and clinical observations and high renal damage risk documented in Fischer 344 rat toxicity studies and clinical reports, it has been largely eliminated from clinical use (14). In practical application, due to its potential side effects and safety issues, clinicians tend to choose other drugs with higher safety for treatment.

### 2.4 Lesinurad

Lesinurad is a selective urate transporter 1 (URAT1) inhibitor. The clinical application of lesinurad mainly focuses on combination with xanthine oxidase inhibitors (such as allopurinol or febuxostat) to improve the uric acid-lowering effect. Clinical trials have shown that lesinurad has a significant effect in reducing serum uric acid levels, but its efficacy is limited when used alone (15). The safety of lesinurad has also attracted much attention, especially the possible acute kidney injury. Studies have shown that the incidence of renal safety events of lesinurad is 11.2%, which has led to its withdrawal from the markets in the United States and Europe (16). In that study, the authors conducted a systematic review of randomized controlled trials and then developed a semi-mechanistic pharmacokinetic/pharmacodynamic (PK/PD) model to characterize the dose–exposure–effect relationship of several URAT1 inhibitors(including lesinurad, verinurad, dotinurad and SHR4640), both as monotherapies and in combination with XOIs. Studies have shown that Verinurad (RDEA3170), an iterative drug of lesinurad, has significantly improved efficacy and safety. The affinity of Verinurad for URAT1 is 170 times that of lesinurad. A daily dose of 20 mg can reduce serum uric acid by 327 μmol/L compared with the baseline. A phase IIa trial showed that when combined with 80 mg febuxostat to treat asymptomatic hyperuricemia, the improvement rate of proteinuria reached 42%, and no acute kidney injury was observed (17).

### 2.5 Tranilast

Tranilast is originally an etiological therapeutic drug for allergic diseases. It through *in vitro* experiments using HEK293 cells stably expressing human URAT1, GLUT9, SLC17A1, OAT1 or OAT3 and it inhibits URAT1 and GLUT9-mediated urate transport in a completely reversible non-competitive manner, and inhibits the secretory urate transporters SLC17A1 (Human sodium-dependent phosphate cotransporter type 1), OAT1, and OAT3 to play its role. In recent years, studies have been conducted to develop tranilast-based derivatives with stronger uric acid-lowering effects. These derivatives have dual inhibitory activity, which indicates that they may reduce serum uric acid levels through two different mechanisms: on the one hand, they reduce the reabsorption of uric acid in the kidney by inhibiting URAT1, and on the other hand, they reduce the production of uric acid by inhibiting Xanthine Oxidase (XOD) (18). However, due to extensive drug-transporter interactions characterized in cellular models and clinical observations and high renal damage risk documented in animal toxicity studies and clinical reports, the parent drug tranilast has been largely eliminated from clinical use. Future studies need to further explore the pharmacokinetic characteristics, safety evaluation, and long-term therapeutic effects of tranilast derivatives to verify their feasibility and effectiveness as drugs for the treatment of hyperuricemia. At the same time, the structural optimization strategy based on tranilast also provides a useful reference for the modification of other drugs and the development of new drugs.

### 2.6 Research and development of URAT1 inhibitors

Among the current drugs that promote uric acid excretion for the treatment of hyperuricemia, the URAT1 inhibitor SHR4640 has achieved significant research and development results: Verified by subHUA (24-hour subacute hyperuricemia) and Ch-HUA (21-day chronic hyperuricemia) mouse models, its 50 mg/kg dose can effectively reduce uric acid in both models (serum uric acid decreased by 42% 3 days after Ch-HUA administration), and long-term administration causes no renal damage. In addition, CC18002 and CC17001 were selected from 22 candidate compounds (the uric acid-lowering effect of 50 mg/kg dose is equivalent to that of the same dose of benzbromarone). This dual-model also provides an accurate tool for the dose optimization and safety evaluation of domestic URAT1 inhibitors. In a phase IIb trial in South Korea, the 3–10 mg dose can achieve a maximum serum uric acid reduction of 46% (Emax), which is significantly better than that of benzbromarone (23%), and the risk of fulminant hepatitis is lower than that of traditional uric acid excretion-promoting drugs. At present, it has obtained clinical implied permission in China (19).

## 3 Research progress of uric acid-lowering drugs inhibiting uric acid synthesis

**Figure 2:**
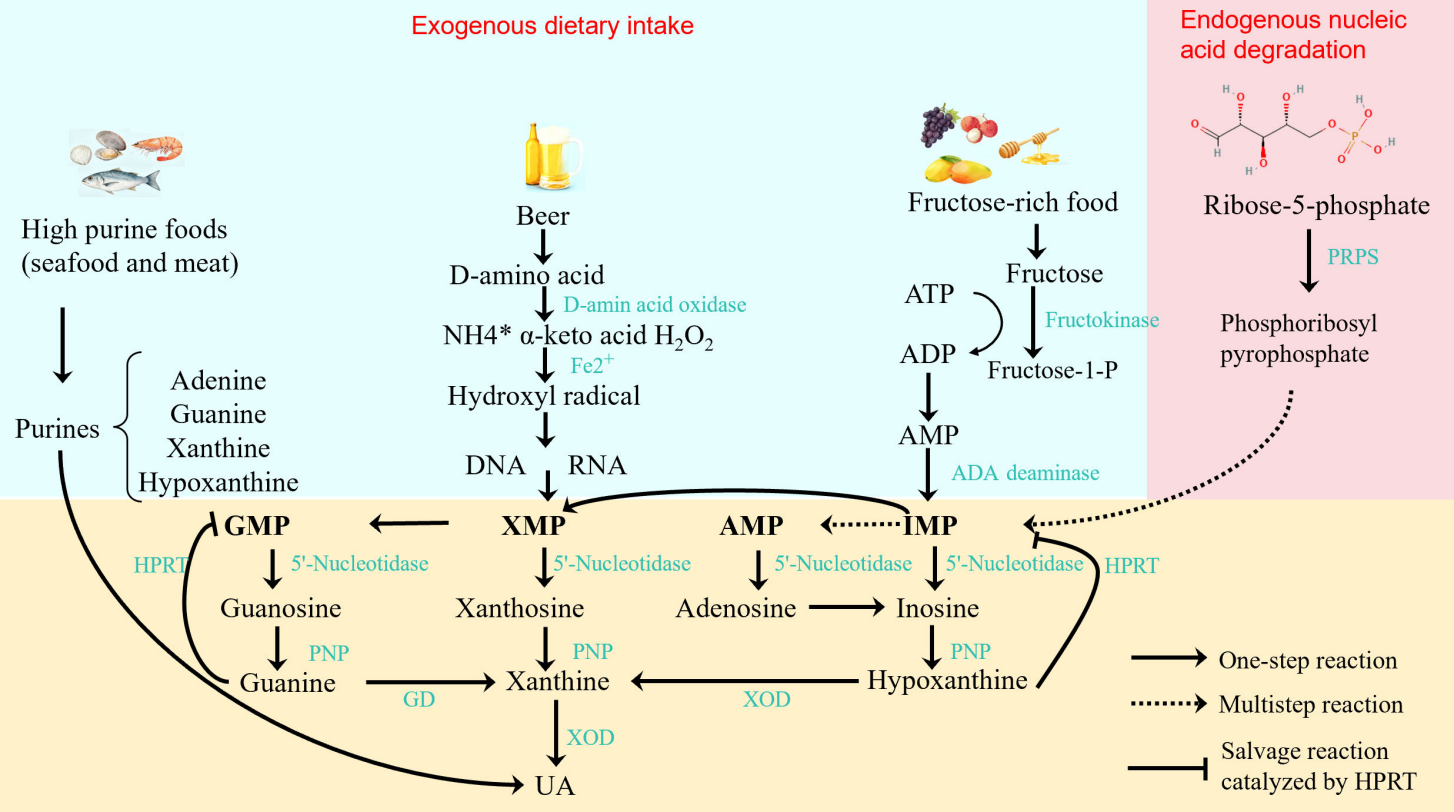
Schematic diagram of purine metabolism pathway and uric acid generation mechanism.

Xanthine oxidoreductase (XOR), as a part of the molybdenum-containing dehydrogenase flavoprotein family, plays a key rate-limiting enzyme role in purine metabolism in the body. It is composed of two mutually convertible forms: xanthine dehydrogenase (XDH) and xanthine oxidase (XOD). These two enzymes convert hypoxanthine and xanthine into uric acid in the final step of purine metabolism, while producing reactive oxygen species (ROS). The activity of XOR is regulated by a variety of factors, including the structure of the enzyme itself, gene expression regulation, and a variety of endogenous and exogenous compounds (20). In the research of drugs for the treatment of hyperuricemia, inhibiting the activity of XOR is a key strategy to reduce serum uric acid levels. Purine metabolism, a critical component of the body’s nucleic acid metabolism, involves a sequential enzymatic pathway where purine precursors—from endogenous nucleotide (AMP, IMP, XMP, GMP) degradation and exogenous high-purine food (e.g., seafood, meat) intake—are converted to uric acid: 5’-Nucleotidase first hydrolyzes these nucleotides into adenosine, inosine, xanthosine, and guanosine; adenosine deaminase converts adenosine to inosine, while purine nucleoside phosphorylase breaks down nucleosides into hypoxanthine, xanthine, and guanine (guanine is further converted to xanthine by guanine deaminase); finally, XOR (existing as interconvertible XDH and XOD, a key rate-limiting enzyme) oxidizes hypoxanthine and xanthine to uric acid, with ROS generated during this process. Humans lack functional uricase (preventing uric acid degradation to excretable allantoin), so uric acid is the final purine metabolite; abnormal elevation of serum uric acid (exceeding 420 μmol/L) occurs via excessive production (e.g., hyperactive purine metabolic enzymes), impaired excretion, or high-purine intake, leading to hyperuricemia and increased risks of gout, kidney damage, and cardiovascular diseases. Key enzymes (notably XOR) and transporters (e.g., URAT1, GLUT9) in this pathway are core targets for clinical uric acid-lowering drugs. The schematic diagram of purine metabolism pathway and uric acid generation mechanism is as shown in the **Figure 2**.

### 3.1 Allopurinol

Allopurinol is a classic uric acid-lowering drug launched in the 1960s. It reduces uric acid production by inhibiting XOD activity, and its main metabolite oxypurinol also has XOD inhibitory effect (21). The efficacy and safety of this drug have been verified in a number of studies, especially in elderly patients and patients with heart failure and cancer, it has good tolerance (22). It should be noted that allopurinol has some non-negligible adverse reactions, such as skin allergic reactions and drug-induced liver and kidney function damage. The identified risk factors include race, HLA-B*5801 genotype, renal insufficiency, initial dose, and combined use of diuretics (23). To reduce these risks, it is recommended to screen HLA-B*5801 in high-risk individuals, start allopurinol treatment with a low dose, and educate patients to identify the signs and symptoms of severe skin adverse reactions and how to deal with them (24). At present, allopurinol is still the first-line drug for the treatment of hyperuricemia, especially in long-term treatment, its low cost and good safety give it significant advantages.

### 3.2 Febuxostat

Febuxostat is a second-line uric acid-lowering drug, which is a new type of selective non-competitive xanthine oxidase inhibitor. It has an effective uric acid-lowering effect and can be used as a substitute for patients who are intolerant to allopurinol. Moreover, it can be used in patients with poor renal function. The results of a RCT study showed that febuxostat is safe for patients with GFR of 15 ml/min (25). The cardiovascular safety of febuxostat has attracted extensive attention. In February 2019 (26), the U.S. Food and Drug Administration (FDA) issued a safety communication stating that febuxostat (Uloric) was associated with an increased risk of heart-related death compared with allopurinol, based on results from the CARES trial. Consequently, a boxed warning was added to the drug label to alert prescribers of this cardiovascular risk. However, subsequent meta-analyses have suggested that long-term use of febuxostat is not associated with an increased risk of all-cause or cardiovascular mortality when compared with allopurinol (27). In recent years, progress has been made in the research of febuxostat and its derivatives. Different parts of its structure, such as substituted benzene rings and carboxyl-substituted heterocycles, have an important impact on its activity of inhibiting XOD. Through structural modification, it is expected to design and develop febuxostat derivatives with better uric acid-lowering effects (28). In recent years, studies have found that due to the anti-inflammatory and antioxidant properties of febuxostat, it also has potential for multi-aspect treatment and can be applied in kidney protection and osteoarthritis control (29). For example, a RCT study showed that febuxostat can reduce the risk of occurrence and deterioration of massive albuminuria (30). Future studies will evaluate the long-term efficacy and safety of febuxostat in more patient populations, and may develop a new generation of xanthine oxidase inhibitors based on its structural optimization to provide more options for the treatment of hyperuricemia. In-depth understanding of its mechanism of action will promote the precise clinical application of febuxostat.

### 3.3 Topiroxostat

Topiroxostat is a new type of selective xanthine oxidase inhibitor. As a mixed-type inhibitor, topiroxostat not only binds to the hydrophobic cavity of the enzyme, but also forms a covalent bond with the molybdopterin center. The dual mechanism of action enhances its inhibitory effect. A cohort study showed that 160 mg/d topiroxostat can reduce serum uric acid by 44.8% in patients with hyperuricemia complicated with chronic heart disease during pregnancy, and has no significant impact on fetal heart rate and amniotic fluid volume; For patients with stage 3 chronic kidney disease with GFR of 15–30 ml/min, this drug can maintain the annual increase of Scr < 5%, and its renal function stability is better than that of febuxostat (the annual increase of Scr is 8.2%) (30). Clinical studies have shown that topiroxostat can not only effectively reduce serum uric acid levels, but also has cardioprotective and nephroprotective effects and potential for weight regulation: In a diabetic rat model, topiroxostat can reduce oxidative stress and protect aortic function (31). A clinical study showed that topiroxostat may have a positive impact on the level of brain natriuretic peptide (BNP) in patients with heart failure with preserved ejection fraction (32). In terms of renal protection, a cohort study showed that topiroxostat can improve the renal function of patients with hyperuricemia (33). A study by Hagiwara et al. further confirmed that it can also alleviate early renal pathological changes in a type 2 diabetic mouse model (34). In addition, topiroxostat can inhibit weight gain without affecting food intake. Further studies have shown that it promotes the body to enter a catabolic state by promoting lipid combustion and activating the salvage pathway, which may further promote weight loss (35).

## 4 Research progress of uric acid-lowering drugs promoting uric acid hydrolysis

Recombinant uricases, such as rasburicase and pegloticase, are biological agents used for the treatment of refractory gout and hyperuricemia. Their mechanism of action is to convert uric acid into more easily excreted allantoin, thereby rapidly and effectively reducing uric acid. Rasburicase and pegloticase have their own unique advantages and indications in the treatment of hyperuricemia. Uricase drugs can play an important role in the treatment of gout, for example, as a treatment option for patients with severe tophi, which can be used for a short period of several months to quickly dissolve tophi (36). However, due to safety risks caused by immunogenicity (such as the production of anti-drug antibodies and infusion reactions) and the fact that they have not been marketed in China, their clinical application is limited. A comprehensive comparison of commonly used Western urate-lowering agents is summarized in **Table 1**.

**Table 1:** Comparison of commonly used western medicines for lowering uric.

Drug category	Drug name	Target of action	Key mechanism	Major safety concerns	Core information on approval by FDA, NMPA, and EMA	Ref.
Uric Acid Excretion-Promoting Drugs	Benzbro-marone	URAT1/ GLUT9	Competitive inhibition of renal urate reabsorption	Hepatotoxicity	Not approved by FDA or NMPA; withdrawn in EU due to hepatotoxicity	(5, 8)
	Dotinurad	URAT1 (high selectivity)	Selective inhibition of URAT1	Generally safe; monitor renal function	Approved in Japan and China (2024); not approved by FDA or EMA	(10, 11)
	Probenecid	URAT1/OATs (non-selective)	Non-selective inhibition of urate/OAT transport	Renal toxicity; drug interactions	Approved by FDA (since 1979); not approved for gout by EMA or NMPA	(13, 14)
	Lesinurad	URAT1	Add-on therapy with XOD inhibitors	Acute kidney injury; withdrawn from US/EU	Approved by FDA (2015) and EMA (2016); withdrawn from both markets	(15, 16)
	Verinurad, an iterative drug of Lesinurad	URAT1	High-affinity URAT1 inhibition	GI effects; renal AEs reported	Not approved by any agency; in clinical development globally	(17)
	Tranilast	URAT1, GLUT9, SLC17A1, OAT1/3	Dual inhibition of reabsorption and secretion	GI reactions; limited clinical use	Approved in China (for allergy/asthma); not approved for hyperuricemia by FDA or EMA	(18)
Uric Acid Synthesis-Inhibiting Drugs	Allopurinol	XOD	Inhibition of hypoxanthine → uric acid conversion	Hypersensitivity; hepatic/renal toxicity	Approved by FDA, EMA, and NMPA; widely used as first-line agent	(22, 23)
	Febuxostat	XOD (non-purine)	Selective inhibition of XOD	CV safety controversy	Approved by FDA (2009), EMA (2008), and NMPA; boxed warning for CV risk	(25, 30)
Uric Acid Hydrolysis-Promoting Drugs	Topiroxostat	XOR (mixed-type)	Inhibits both XDH and XOD forms	Good renal safety; metabolic effects	Approved only in Japan; not approved by FDA, EMA, or NMPA	(31, 34)
	Pegloticase	Uricase	Converts uric acid → allantoin	Immunogenicity; infusion reactions	Approved by FDA (2010); withdrawn from EMA; not approved by NMPA	(37, 38)
	Rasburicase	Uricase	Rapid uric acid oxidation (TLS use)	Hemolysis (G6PD deficiency)	Approved by FDA, EMA, and NMPA for tumor lysis syndrome–related hyperuricemia	(39, 40)

### 4.1 Pegloticase

Pegloticase is a recombinant urate oxidase used for the treatment of refractory gout. It catalyzes the oxidation of uric acid into 5-hydroxyisouric acid and hydrogen peroxide, which are further hydrolyzed and decarboxylated to form more soluble metabolites allantoin, which are finally excreted through the kidneys, thereby reducing serum uric acid levels. It is mainly used for the treatment of chronic gout patients who are ineffective or intolerant to conventional treatment. In clinical trials, pegloticase has shown significant effects in reducing serum uric acid levels and relieving joint pain. The efficacy of pegloticase is limited by its immunogenicity, which may lead to the production of anti-drug antibodies, thereby increasing drug clearance, reducing efficacy, and possibly causing infusion reactions (37). There are clear quantitative data on the tophi dissolution effect of pegloticase: A multicenter study showed that intravenous injection of 8 mg pegloticase every two weeks can reduce the volume of tophi by more than 70% within 13 weeks, and the complete dissolution rate of foot tophi reaches 38%; For patients with end-stage renal disease undergoing dialysis, hemodialysis does not affect the drug concentration, and the uric acid-lowering effect is equivalent to that of non-dialysis patients (38). A special safety issue to note is that patients with glucose-6-phosphate dehydrogenase (G6PD) deficiency should avoid using it or be screened before use, because red blood cells are sensitive to oxidants such as hydrogen peroxide, which may lead to hemolytic anemia and methemoglobinemia (41).

### 4.2 Rasburicase

Rasburicase is a recombinant urate oxidase, mainly used to rapidly reduce the level of uric acid in the blood, especially for patients with tumor lysis syndrome (TLS) (38). Clinical guidelines recommend that for patients with acute lymphoblastic leukemia and lymphoma undergoing chemotherapy, preventive use of rasburicase (0.2 mg/kg, qd × 3d) can reduce the incidence of hyperuricemia from 45% to 12% without adjusting the chemotherapy dose, and can reduce the risk of acute kidney injury and death caused by chemotherapy-induced hyperuricemia (39). Similar to pegloticase, when patients with G6PD deficiency use rasburicase, the sensitivity of red blood cells to oxidative stress of hydrogen peroxide increases, which can cause hemolytic anemia and methemoglobinemia (40).

## 5 Research progress of Traditional Chinese Medicine drugs and therapies for hyperuricemia

At present, most Western medicines for lowering uric acid have some adverse reactions, such as allergic reactions and liver function damage of allopurinol, and controversy over the cardiovascular safety of febuxostat. However, in recent years, with the development of Traditional Chinese Medicine (TCM), new methods such as syndrome differentiation and constitutional toxicology can be used to evaluate the safety of TCM (42). Therefore, TCM has gradually become a research focus in the field of uric acid lowering, mainly including single herbs and their monomers, TCM compound prescriptions, and external TCM therapies.

### 5.1 Single herbs and their monomers for lowering uric acid

#### 5.1.1 Inhibition of uric acid production

XOD is a key enzyme in the *de novo* synthesis of uric acid. A variety of single herbs and their monomers can reduce uric acid production by inhibiting XOD activity: Zhu Jixiao and other researchers found that geniposide and crocin-I in Gardenia jasminoides can inhibit XOD activity (43). Perilla frutescens extract (44) and Salvia miltiorrhiza extract (45) can also inhibit XOD activity. Wang Jinpiao and others confirmed in animal experiments that the ethanol extract of Alisma orientale can reduce the serum uric acid level of hyperuricemic rats by reducing XOD activity (46). Luteolin effectively reduces the catalytic activity of XOD by competitively and reversibly inhibiting the construction of the active center of XOD, and its inhibitory effect is better than that of allopurinol (47). Evodiamine in Evodia rutaecarpa can reduce the serum uric acid level of hyperuricemic quails by inhibiting the activities of XOD and guanine deaminase (GD) (48).

#### 5.1.2 Regulation of uric acid transport

Renal uric acid transporters are the core regulatory targets for uric acid excretion. Some single herbs can affect uric acid excretion by regulating the expression of transporters: Fraxinus rhynchophylla can reduce the serum uric acid of hyperuricemic rats by inhibiting two uric acid reabsorption proteins, URAT1 and GLUT9, and can also alleviate kidney damage caused by hyperuricemia and reduce urinary creatinine level. The active component saponin extracted from Dioscorea opposita can up-regulate the expression of OAT1 and OAT3, and down-regulate the expression of URAT1 and GLUT9, thereby reducing the uric acid of hyperuricemic mice (49). Studies have shown that the ethanol extract of Eucommia ulmoides cortex can significantly increase the mRNA expression of organic OAT1 and OAT3 in the kidneys of hyperuricemic rats, and at the same time significantly reduce the mRNA levels of GLUT9 and urate transporter 1 (URAT1), which proves its potential role in improving hyperuricemia (49). Representative Traditional Chinese Medicines and their active monomers with urate-lowering activity are listed in **Table 2**. Bergenin can regulate the excretion of serum uric acid in the body by regulating the expression levels of ATP-binding cassette subfamily G member 2(ABCG2) and GLUT9, and at the same time, it can reduce the inflammatory response in the body, so it has high potential in the treatment of hyperuricemia and its complications (51).

**Table 2:** Representative Traditional Chinese Medicines/monomers with uric acid-lowering activity.

Category	Name of TCM/monomer	Active component	Mechanism of action	Research model	Ref.
I. Mainly Inhibiting Uric Acid Production	Gardenia jasminoides	Geniposide, Crocin-I	Inhibition of XOD	Potassium oxonate-induced HUA rat model	(43)
	Perilla frutescens	Rosmarinic acid (main)	Blocks XOD-mediated purine oxidation	Potassium oxonate-induced HUA mouse model	(44)
	Salvia miltiorrhiza	Tanshinone IIA	Down-regulates hepatic XOD	Potassium oxonate-induced HUA rat model	(45)
	Alisma orientale	Alisol B	Inhibits XOD; reduces purine intermediates	Potassium oxonate-induced HUA rat model (14-day administration)	(46)
	Fraxinus rhynchophylla	Fraxin	Inhibits URAT1/GLUT9; reduces renal crystals	Potassium oxonate-induced HUA rat model (14-day administration)	(49)
II. Mainly Regulating Uric Acid Transport	Dioscorea opposita	Dioscorea saponin	↑OAT1/OAT3; ↓URAT1/GLUT9	Potassium oxonate-induced HUA mouse model	(50)
	Eucommia ulmoides	Eucommioside	Modulates renal transporters	Potassium oxonate-induced HUA rat model (21-day administration)	(49)
	Bergenia purpurascens	Bergenin (monomer)	↑ABCG2; ↓GLUT9; anti-inflammatory	Potassium oxonate-induced HUA mouse model	(51)
III. Multi-Mechanism Synergistic Action	Smilax glabra	Smilax glabra saponin	Inhibits XOD; ↓URAT1/GLUT9; anti-inflammatory	Potassium oxonate-induced HUA rat model	(50)
	Fibraurea recisa	Palmatine (monomer)	↓URAT1/GLUT9; ↑OAT1/ABCG2; inhibits XDH/ADA	Potassium oxonate-induced HUA mouse model	(52)
	Scutellaria baicalensis	Baicalein (monomer)	Non-competitive inhibition of URAT1/GLUT9 & XDH	Potassium oxonate-induced HUA rat model	(53)
	Hirudo nipponica	Hirudin	Inhibits XOD; ↓GLUT9	Potassium oxonate-induced HUA mouse model	(54)
IV. Flavonoids (Clear Structure-Activity Relationship)	Quercetin (flavonol)	–	Anti-XOD; antioxidant	Potassium oxonate-induced HUA mouse model	(55)
	Galangin (flavone)	–	Inhibits XDH	Potassium oxonate-induced HUA rat model	(56)
	Apigenin (flavone)	–	↓URAT1/GLUT9; ↓fibrosis	Potassium oxonate-induced HUA mouse model	(57)
	Chrysin (flavone)	–	Inhibits XDH; modulates renal transporters	Urate crystal-induced gouty arthritis rat model	(58)

#### 5.1.3 Multi-mechanism synergistic uric acid lowering

Some single herbs can play a role in lowering uric acid through the dual mechanisms of inhibiting hepatic uric acid synthesis and promoting renal and intestinal uric acid excretion. Smilax glabra can inhibit the activity of hepatic XO, and at the same time down-regulate GLUT9 and URAT1 to promote renal uric acid excretion. While reducing the body’s uric acid, it can also inhibit the inflammatory factors IL-1B and TNF-α, thereby playing a role in protecting the kidney (50). Liu Xihua and others found that hirudin in Hirudo nipponica can inhibit XOD activity and change the expression of GLUT9, thereby reducing the uric acid of mice from both aspects of inhibiting uric acid synthesis and promoting uric acid decomposition (54). Dianthus chinensis and Polygonatum odoratum also showed uric acid-lowering effects in hyperuricemic mice modeled by the combination of potassium oxonate and hypoxanthine. The mechanism may be related to down-regulating the protein expressions of GLUT9 and URAT1, up-regulating the protein expression of OAT1, and inhibiting XOD activity, so as to achieve the effect of lowering uric acid (59). Baicalein inhibits GLUT9 and URAT1 in a non-competitive and dose-dependent manner, and can also inhibit the activity of xanthine dehydrogenase (XDH), thereby synergistically reducing serum uric acid through multiple targets (53). Studies have shown that palmatine can significantly down-regulate the protein levels of GLUT9 and URAT1 in hyperuricemic mice, and at the same time up-regulate the protein expressions of OAT1 and ABCG2. It can also significantly reduce the activities of XDH and adenosine deaminase (ADA) in the liver, showing a strong uric acid-lowering effect; In addition, palmatine can also reduce the kidney damage induced by HUA by restoring the Keap1-Nrf2 pathway and inhibiting the TXNIP/NLRP3 inflammasome (52). Further studies have found that its metabolite 9-OPAL (9-Hydroxy-8-oxypalmatine) can continue the uric acid-lowering effect of palmatine (60).

#### 5.1.4 Uric acid-lowering effect of flavonoids

Flavonoids are the most studied natural compounds in the field of hyperuricemia treatment at present (61). Their inhibitory effects on XOD vary due to different structures, among which the inhibitory activity of flavones on xanthine dehydrogenase (XDH) is better than that of flavonols (62). Quercetin is a flavonol compound with a variety of biological activities and high medicinal value. Studies have shown that its molecular structure is complementary to the active site of XOD, which can prevent the substrate xanthine from entering the active center of XOD, thereby inhibiting the activity of XOD and reducing the production of uric acid. In animal experiments, quercetin can effectively reduce the serum uric acid level of hyperuricemic mice without damaging renal function (55). Galangin can inhibit the activity of XDH and play its role in lowering uric acid (56). Apigenin, as a kind of flavonoid compound, has lower toxicity than quercetin. It can regulate the expressions of mURAT1, mOCTN1, mOCTN2, mOCT1, and mOCT2, reduce the serum uric acid of mice, and improve renal fibrosis. It has significant value in the treatment of hyperuricemia and uric acid nephropathy (57). Chrysin also belongs to flavonoid compounds and has the effect of inhibiting XDH activity. At the same time, it can promote uric acid excretion by regulating the protein levels of OAT1, ABCG2, URAT1, and GLUT9 in the kidney, and can also treat gouty arthritis by affecting oxidative stress and inflammatory response (58).

### 5.2 TCM compound prescriptions for lowering uric acid

TCM has a long history in the treatment of hyperuricemia and gout. Classic compound prescriptions and improved prescriptions are widely used in clinical practice: Simiao Wan, composed of Phellodendron chinense, Atractylodes lancea, Achyranthes bidentata, and Coix lacryma-jobi, has been used for the treatment of gout and gouty arthritis for more than 700 years; The modified Jiawei Simiao Wan based on Simiao Wan can play a role in lowering uric acid by inhibiting XOD activity and regulating the expressions of URAT1 and OAT1 (63). Qingre Chubi Fang has also been proved to be able to alleviate gouty arthritis by inhibiting the expression of IL-1β (64). Cai Tangyan and other researchers found that Tongfengning can affect the expressions of uric acid transporters such as URAT1, GLUT9, ABCG2, and OAT1, and promote the renal excretion of uric acid (65). Kaempferol is a flavonoid compound. Studies have shown that it inhibits the activity of XOD by interacting with the hydrophobic cavity of XOD and occupying its active site, thereby reducing the production of uric acid.

### 5.3 External TCM therapies

External TCM therapies regulate serum uric acid levels through multiple mechanisms and show unique advantages in the treatment of hyperuricemia: Filiform needle acupuncture and acupuncture at specific acupoints can reduce uric acid production and alleviate inflammation by reducing XOD activity and inhibiting the expression of inflammatory factors (66, 67). In addition, the “Tongjing Lizhuo” acupuncture method can reduce the inflammatory response by inhibiting the activation of the NF-kB signaling pathway at the molecular level (68). Other external therapies such as bloodletting therapy with pricking, cupping, and bleeding can reduce the inflammatory factors in the blood and enhance the expression of local anti-inflammatory factors, thereby jointly alleviating the inflammation caused by hyperuricemia. Clinical results show that it can reduce the erythrocyte sedimentation rate (ESR), C-reactive protein (CRP), and uric acid levels without adverse events. These studies further confirm the multi-pathway and multi-target effects of external TCM therapies in regulating the body’s immune response and metabolic process. These treatment methods provide effective treatment options for hyperuricemia and its related inflammatory diseases, and show the practical value and scientificity of TCM in modern medical treatment. A 2024 Meta-analysis evaluated the clinical efficacy of different acupuncture therapies in lowering uric acid. By screening 32 randomized controlled trials (RCTs), a total of 2434 patients with acute gouty arthritis (AGA) were included. The results showed that acupoint application was the best in improving the Visual Analogue Scale (VAS) score for pain, increasing the total effective rate, and reducing the serum uric acid (SUA) level; Acupuncture has advantages in reducing the erythrocyte sedimentation rate (ESR) and reducing adverse events, which confirms that external TCM therapies can be used as effective treatment options for hyperuricemia (69).

### 5.4 Integrative comparison between western and Traditional Chinese Medicine approaches

In the treatment of hyperuricemia, Western medicine and TCM represent distinct yet complementary paradigms. Western pharmacotherapy emphasizes precise molecular targeting, primarily by inhibiting key enzymes such as xanthine oxidase (XOD) or transporters such as URAT1, and by enhancing uric acid degradation to achieve rapid serum uric acid reduction with well-defined efficacy and dosage control (4). In contrast, TCM approaches the disease from a holistic regulatory perspective, considering it as a disorder of internal imbalance such as damp-heat accumulation or spleen deficiency. TCM focuses on clearing heat and removing dampness and strengthening the spleen to eliminate turbidity, utilizing multi-component, multi-target, and multi-pathway mechanisms to improve metabolism and inflammation, thereby achieving long-term homeostasis (63).

Mechanistically, Western drugs often act on single targets, offering rapid efficacy but with a higher risk of adverse reactions. In contrast, TCM monomers and formulae exert multitarget synergistic effects—simultaneously inhibiting XOD activity, modulating renal urate transporters (URAT1, OAT1, ABCG2), and alleviating oxidative stress and inflammatory responses by regulating pathways such as NLRP3 and NF-κB (53). These characteristics endow TCM with advantages in maintaining metabolic stability and renal protection. Recent studies have shown that although herbal and acupuncture therapies produce a slower urate-lowering response, they are associated with better safety and tolerance, and can effectively improve chronic inflammation and metabolic disorders (69). Therefore, TCM represents a promising adjunctive or long-term maintenance option.

Looking forward, integrative therapeutic strategies combining Western and TCM approaches merit further exploration. For instance, using low-dose XOD inhibitors in combination with flavonoid-rich herbal formulations may enhance efficacy while reducing hepatotoxicity. Similarly, incorporating probiotics or washed microbiota transplantation (WMT) can modulate uric acid metabolism through the gut–kidney axis. By integrating modern pharmacology, multi-omics, and metabolomic technologies under the guidance of the Toxicological Evidence Chain (TEC) concept proposed by Liu (70–72), the pharmacological and safety transmission mechanisms of TCM *in vivo* can be systematically elucidated. Through the comprehensive integration of Clinical Risk Evidence (CRE), Harmful Ingredient Evidence (HIE), Injury Phenotype Evidence (IPE), Toxic Event Evidence (TEE), and Adverse Outcome Evidence (AOE), this approach facilitates the establishment of a multidimensional TCM intervention framework that balances efficacy and safety, thereby promoting the precision, safety, and multidimensional development of TCM-based therapy for hyperuricemia.

## 6 Research progress of other uric acid-lowering drugs or methods

### 6.1 SGLT2 inhibitors

SGLT2 inhibitors are a class of drugs used for the treatment of type 2 diabetes. They reduce the reabsorption of glucose by inhibiting the sodium-glucose cotransporter 2 (SGLT2) in the kidney, thereby increasing the excretion of glucose in the urine and lowering blood glucose levels. In recent years, studies have shown that SGLT2 inhibitors show significant effects in reducing serum uric acid levels and reducing the frequency of gout attacks. In clinical experiments, compared with the fasting state (average 5.3 ± 1.1 mg/dl), dapagliflozin further reduced serum uric acid by 0.2 ± 0.3 mg/dl and 0.4 ± 0.3 mg/dl in the hyperinsulinemia and hyperglycemia states, respectively (73). Further exploration of its mechanism shows that SGLT2 inhibitors regulate uric acid metabolism through two pathways: on the one hand, they simulate the starvation state, reduce the flux of the pentose phosphate pathway, and reduce the synthesis of purines and uric acid; on the other hand, they increase renal uric acid excretion, including increasing the activity of GLUT9 in the proximal convoluted tubule and possibly up-regulating ABCG2. ABCG2 is an important uric acid transporter, which is responsible for transporting uric acid from the blood to the renal tubule lumen, thereby promoting the excretion of uric acid (74, 75). In addition, SGLT2 inhibitors can reduce the hospitalization rate of patients with heart failure and protect the kidney. Therefore, in patients with type 2 diabetes, reducing uric acid by inhibiting SGLT2 may help reduce adverse cardiovascular events and delay the progression of chronic kidney disease (CKD) (76).

### 6.2 GLP-1/GCG dual-receptor agonist mazdutide

Mazdutide is a once-weekly glucagon-like peptide 1 (GLP-1)/glucagon (GCG) receptor dual agonist. Its unique dual-receptor synergistic mechanism enables it to show significant advantages in the field of uric acid lowering. In recent years, both clinical and basic studies have confirmed its uric acid-lowering efficacy, and revealed its multi-target regulatory pathway of uric acid metabolism at the molecular level: In a phase 1b study of obese people, it was found that overweight and obese patients treated with 4.5 mg and 6.0 mg Mazdutide had an average reduction of serum uric acid (SUA) by 83.47 μmol/L and 87.48 μmol/L, respectively, at week 12 of administration, and the difference was statistically significant compared with the placebo group (77). In the phase 2 study of Mazdutide in Chinese obese people, SUA was set as a secondary efficacy endpoint for re-evaluation as a risk factor for cardiovascular metabolism. The results showed that Mazdutide at doses of 3–6 mg could significantly reduce the SUA level after 24 weeks of intervention, and the level decreased by an average of 48.6-72.6 μmol/L compared with the placebo group, which was statistically significant (78). Later, our team conducted a further in-depth study on rats with hyperuricemia induced by modeling. After 18 days of Mazdutide intervention, the reduction of serum uric acid level was equivalent to that of allopurinol, while there was no change in the semaglutide group. Other biochemical parameters also showed changes to varying degrees. At the same time, Mazdutide intervention could improve renal pathology and regulate oxidative stress (79). Subsequent transcriptomic results showed that Mazdutide could reduce the precursor substances of uric acid production by regulating the expressions of genes such as GCGR, Slc22a7, Slc23a3, Aqp2, Dnmt3a, Rest, Foxn3, Atp7a, and Slc4a7, and play a role in reducing serum uric acid by affecting glucose and lipid metabolism, purine metabolism, and bile secretion (80). At present, the results of a single-center, randomized, double-blind, placebo-controlled clinical study (protocol number CIBI362Y001) on the efficacy and safety of Mazdutide in obese or overweight patients with hyperuricemia (HUA) still confirm the previous conclusions.

The latest team study found that there is a temporal correlation between the uric acid-lowering effect of Mazdutide and weight regulation: *Post-hoc* analysis showed that in obese hyperuricemia patients treated with Mazdutide, for every 1 kg/m² decrease in body mass index (BMI), the serum uric acid decreased by an average of 12.6 μmol/L, and the compliance rate of serum uric acid (68%) in patients with weight loss in the first 8 weeks was significantly higher than that in the group with delayed weight loss (32%), suggesting that weight loss plays an auxiliary role in its uric acid-lowering effect. At present, the team is focusing on deepening the mechanism exploration to provide a more accurate mechanism basis for clinical individualized medication (81).

### 6.3 Probiotics

In recent years, the role of intestinal flora in uric acid metabolism has been gradually clarified. Studies have shown that probiotics can play a role in lowering uric acid through multiple pathways: producing active substances to reduce XOD activity, generating uricase to degrade uric acid, regulating intestinal uric acid transporters to increase uric acid excretion, and improving intestinal barrier permeability to reduce chronic inflammation (82, 83). A study found that the Limosilactobacillus fermentum JL-3 strain isolated from traditional fermented foods can secrete uricase, which can significantly reduce the serum uric acid level by up to 31.3% in *in vitro* experiments, and is expected to improve hyperuricemia. Some Lactobacillus strains can produce intracellular uricase in the gastrointestinal tract and adapt to the gastrointestinal environment to maintain activity; Lactobacillus gasseri PA-3s can metabolize the intermediate products of high-purine foods and reduce the net absorption of purines in rats (84). The results of such studies show that specific Lactobacillus strains can change the human purine metabolism pathway and slow down the synthesis of uric acid by using ribose or nucleotides to synthesize vitamins such as thiamine, riboflavin, and folic acid (85). In addition, Lactobacillus rhamnosus BFE5264 and Lactobacillus plantarum NR74 can significantly up-regulate the expressions of ABCA1 and ABCG1 at the cellular level. These two transporters are crucial for maintaining uric acid balance. Such findings indicate that probiotics may help prevent and treat hyperuricemia by regulating the activity of uric acid transporters, but their specific mechanisms and effects still need further study (86). Another study found that probiotics can reduce the production of ROS by restoring the abnormal mitochondrial membrane potential, inhibit the activation of the NLRP3 inflammasome, reduce oxidative stress and inflammatory response, and further promote the reduction of uric acid (87). To sum up, probiotics play an important role in regulating uric acid metabolism and reducing the associated inflammation. By remodeling the intestinal flora, the intestinal microecological imbalance of patients with HUA and gout can be improved, thereby reducing the uric acid level. At present, the mechanism by which probiotics improve the intestinal microecological imbalance of patients with HUA and gout by remodeling the intestinal flora still needs in-depth study to optimize their clinical application schemes.

### 6.4 Washed microbiota transplantation

The relationship between hyperuricemia and intestinal dysbiosis has been confirmed. Therefore, washed microbiota transplantation is considered an effective method to restore a healthy intestinal flora. Washed microbiota transplantation can significantly reduce the serum uric acid level of gout patients and reduce the frequency and duration of acute gout attacks. In addition, washed microbiota transplantation can also improve the damaged intestinal barrier function of patients and reduce the levels of biomarkers related to intestinal flora imbalance, such as diamine oxidase (DAO) and endotoxin (88). A retrospective study found that in HUA patients, the serum uric acid level decreased on average after washed microbiota transplantation treatment, and a decrease was observed in 25/32 patients after treatment, and the serum uric acid level returned to normal in 10/32 patients. The change of serum uric acid level before and after treatment showed a moderate correlation. In patients with normal uric acid level (NUA), washed microbiota transplantation had no significant impact on the serum uric acid level, and only 1/144 patients had mild diarrhea after treatment, indicating that washed microbiota transplantation has good safety (89).

### 6.5 Tea beverages

In recent years, studies have found that a variety of traditional tea beverages show potential application value in the field of uric acid lowering due to their rich active components such as flavonoids and polyphenols, and their mechanisms of action focus on different aspects: These tea beverages play a role through a variety of mechanisms, including inhibiting key enzymes in uric acid production, regulating the expression of uric acid transporters, and improving intestinal flora. Ampelopsis grossedentata is a traditional medicinal plant that has been used for the treatment of hyperuricemia and related diseases. Studies have shown that Ampelopsis grossedentata can reduce the production of uric acid by inhibiting the activity of xanthine oxidase (XOD), and may inhibit hyperuricemia by regulating intestinal homeostasis and improving insulin resistance (90). Flavonoids in Ampelopsis grossedentata, such as dihydromyricetin, myricetin, and quercetin, have been proved to have significant uric acid-lowering effects (91). Pu’er tea is a kind of post-fermented tea with a variety of biological activities. The chemical composition of Pu’er tea is complex, including tea polyphenols, tea pigments, catechins, etc. Studies have shown that Pu’er tea and its extracts can inhibit the activity of XOD, reduce the production of uric acid, regulate the expression of uric acid-related transporters, inhibit the reabsorption of urate, and promote the excretion of uric acid (92). The polyphenol oxidation polymers such as theaflavins, thearubigins, and theabrownins in Pu’er tea also show potential in lowering uric acid. Black tea is a fully fermented tea, which contains theaflavins, thearubigins, and other components, and has a variety of biological activities such as treating cardiovascular and cerebrovascular diseases, reducing blood lipid, and losing weight. Studies have found that black tea and its fungus-fermented red brick tea can reduce the serum uric acid level of hyperuricemia model mice, and the mechanism may be related to inhibiting the activities of XOD and ADA (93). Anhua dark tea is a unique microbial fermented tea in China, and its unique processing technology endows it with rich bioactive components. Studies have shown that Anhua dark tea and its active components can effectively reduce the serum uric acid level of hyperuricemia model mice, and its mechanism of action may involve inhibiting key enzymes in uric acid production and regulating the expression of uric acid transporters (94).

## 7 Conclusion and prospect

The core goal of hyperuricemia treatment is to control the serum uric acid level, prevent gout, kidney damage, and cardiovascular complications. As summarized in **Figure 1** and **Tables 1**, **2**, multiple therapeutic strategies targeting uric acid metabolism have been developed, ranging from xanthine oxidase inhibition to transporter modulation and intestinal microecological regulation.

The core goal of hyperuricemia treatment is to control the serum uric acid level, prevent gout, kidney damage, and cardiovascular complications. This review covers the research progress of uric acid-lowering drugs, non-drug therapies, tea beverages, TCM, and emerging treatment methods. Drug treatment includes drugs that promote uric acid excretion (such as benzbromarone and dotinurad), drugs that inhibit synthesis (such as allopurinol and febuxostat), and pegloticase and rasburicase that hydrolyze uric acid. Tea beverages and TCM provide traditional treatment options, while emerging treatment methods such as SGLT2 inhibitors, Mazdutide, probiotics, and washed microbiota transplantation show treatment potential.

Future studies to verify the efficacy and safety of these therapies should focus on three directions: improving the level of evidence, conducting high-quality RCTs on TCM, probiotics, and other therapies to verify their long-term efficacy and safety; promoting mechanism research, clarifying the core targets of action of new therapies such as Mazdutide and WMT, and optimizing drug structures and intervention schemes; individualized treatment, formulating precise treatment strategies based on patient genotypes (such as HLA-B*5801), intestinal flora composition, and mechanisms of uric acid elevation (excessive production/reduced excretion), so as to provide more comprehensive and effective treatment schemes for patients.
